# Supplementary material for: Predicting nutrient content of ray-finned fishes using phylogenetic information
Source: Nat Commun. 2018 Sep 25;9:3742. doi: 10.1038/s41467-018-06199-w (PMC6156416; doi:10.1038/s41467-018-06199-w)
Supplement: Supplementary file 1 — Supplementary Information [file 41467_2018_6199_MOESM1_ESM.pdf]

Supplementary Tables

Supplementary Table 1. List of included species and sources and rich sources of key nutrients. See below for units and thresholds for "source" and "rich source" as designated by FAO.[1] *Values above the source threshold are highlighted in blue, values above the rich source threshold are highlighted in dark blue.*

Nutrient	Source threshold (/100g)	Rich source threshold (/100g)
Protein	7.65 g	15.3 g
Iron	1.95 mg	3.9 mg
Zinc	1.425 mg	2.85 mg
Vitamin A	120 mcg	240 mcg
Vitamin B12	0.75 mcg	1.5 mcg
Vitamin D	0.36 mcg	0.72 mcg

Summary	Protein	Iron	Zinc	Vitamin A	Vitamin B12	Vitamin D
Valid species, n	372	345	178	143	122	122
# Source	17	33	15	7	15	4
# Rich Source	348	11	10	7	99	115
# Source or Rich Source	365	44	25	14	114	119
% Source	4.57	9.57	8.43	4.90	12.30	3.28
% Rich Source	93.55	3.19	5.62	4.90	81.15	94.26
% Source or Rich Source	98.12	12.75	14.04	9.79	93.44	97.54

Species	Common name	Order	Family	Protein	Iron	Zinc	Vit A	Vit B12	Vit D
Acanthistius brasilianus	Argentine Seabass	Perciformes	Serranidae	17.90					
Acanthogobius flavimanus	Goby	Gobioidei	Gobiidae	18.90	2.10	0.60	7	2.70	3.00
Acanthogobius hasta	Javelin goby	Gobioidei	Gobiidae	16.40	0.90				
Acanthopagrus schlegelii	Black sea bream	Perciformes	Sparidae	19.60	0.75	0.80	12	3.70	4.00
Acheilognathus rhombeus	Flat bitterling	Cypriniformes	Cyprinidae	17.50	1.20				
Ailia coila	Gangetic Ailia	Siluriformes	Schilbeidae	15.30	0.90				
Alcichthys alcicornis	Elkhorn sculpin	Scorpaeniformes	Cottidae	17.10	0.80				
Allothunnus fallai	Slender Tuna	Perciformes	Scombridae	19.80	1.30	1.10	227	0.70	
Amblypharyngodon mola	Mola Carplet	Cypriniformes	Cyprinidae	17.10	3.80	3.19	2680		
Ammodytes personatus	Sandlance	Perciformes	Ammodytidae	16.60	7.90	3.90	200	11.00	21.00
Anabas testudineus	Climbing Perch	Perciformes	Abantidae	17.50	1.20	1.13	215		
Anchoa compressa	Deep-bodied anchovy	Clupeiformes	Engraulidae	16.90	1.40				
Anguilla japonica	Eel	Anguilliformes	Anguillidae	15.75	1.05	1.40	2400	3.50	18.00
Anoplopoma fimbria	Sablefish	Scorpaeniformes	Anoplopomatidae	13.35	1.25	0.30	1500	2.80	3.50
Arctoscopus japonicus	Sailfin Sandfish/Sandfish	Perciformes	Trichodontidae	14.10	0.50	0.60	20	1.70	2.00
Argyrops bleekeri	Taiwan tai	Perciformes	Sparidae	17.80	9.10				
Auxis rochei	Frigate mackerel	Perciformes	Scombridae	24.10	1.70				
Auxis thazard	Bullet mackerel	Perciformes	Scombridae	23.40	1.90				
Bagrus bajad	Bayad	Siluriformes	Bagridae	27.50	1.10	0.73	9	2.80	0.80
Banjos banjos	Banjo fish	Perciformes	Banjosidae	20.80	1.40				

Species	Common name	Order	Family	Protein	Iron	Zinc	Vit A	Vit B12	Vit D
Barbonymus gonionotus	Silver Barb	Cypriniformes	Cyprinidae	6.25				2.00	
Belone belone	Needle fish Gar fish Pacific	Beloniformes	Belonidae	19.80	0.50				
Bero elegans	Elegant sculpin	Scorpaeniformes	Cottidae	19.50	0.90				
Beryx decadactylus	Broad alfonsino	Beryciformes	Berycidae	18.50	2.00				
Beryx splendens	Splended Alfonsino	Beryciformes	Berycidae	17.80	0.30	0.30	63	1.10	2.00
Bodianus bilunulatus	Crescent-banded wrasse	Perciformes	Labridae	20.30	1.50				
Boreogadus saida	Polar Cod	Gadiformes	Gadidae		0.25	0.37	24		
Brama japonica	Pacific pomfret	Perciformes	Bramidae	18.90	0.40				
Branchiostegus japonicus	Blanquillo	Perciformes	Branchiostegidae	18.45	0.45	0.30	27	2.10	1.00
Caesio xanthonota	Yellowback Fusilier	Perciformes	Caesionidae	21.10	0.40	0.40	19	0.41	
Callanthias japonicus	Yellowtail red bass	Perciformes	Callanthiidae	17.30	0.70				
Callionymus lunatus	Moon dragonet	Perciformes	Callionymidae	18.50	0.80				
Calliurichthys japonicus	Dragonet	Perciformes	Callionymidae	16.50	1.30				
Carangoides equula	Whitefin trevally	Perciformes	Carangidae	19.60	1.00				
Caranx sexfasciatus	Six-bed jack	Perciformes	Carangidae	24.00	1.70				
Carassius auratus	Crucian carp	Cypriniformes	Cyprinidae	18.15	1.95	1.90	12	5.50	4.00
Catla catla	Catla	Cypriniformes	Cyprinidae	19.90	0.60	0.48	3		0.00
Cephalopholis miniata	Coral Hind	Perciformes	Serranidae	18.40	0.40	0.40	0	1.00	
Channa punctata	Spotted Snakehead	Perciformes	Channidae	17.30	1.50	1.08	191		
Channa striata	Striped Snakehead	Perciformes	Channidae	17.70	1.00	0.31			
Chanodichthys dabryi	Humpback	Cypriniformes	Cyprinidae	18.70	1.10				
Cheilodactylus quadricornis	Black-barred morwong	Perciformes	Cheilodactylidae	19.20	1.30				
Cheilodactylus zonatus	Spottedtail morwong	Scorpaeniformes	Scorpaenidae	19.80	1.30				
Cheilopogon agoo	Japanese flying fish	Beloniformes	Exocoetidae	22.20	0.90				
Chelidonichthys spinosus	Bluefin searobin	Scorpaeniformes	Triglidae	19.75	0.50	0.50	9	2.20	3.00
Chelidoperca hirundinacea	Princess porgy	Perciformes	Serranidae	20.10	1.50				
Chelon parsia	Goldspot Mullet	Mugiliformes	Mugilidae	18.70	2.10	1.43			
Chirolophis japonicus	Fringed blenny	Perciformes	Stichaeidae	17.20	0.60				
Chitala chitala	Clown Knifefish	Osteoglossiformes	Notopteridae	17.80	1.60	0.61	30		
Choerodon azurio	Azurio tuskfish	Perciformes	Labridae	20.30	0.70				
Chromis notata	Pearl-spot chromis	Perciformes	Pomacentridae	19.50	0.70				
Cirrhinus cirrhosus	Mrigal Carp	Cypriniformes	Cyprinidae	18.60	1.80	0.29	11		
Clarias anguillaris	Mudfish	Siluriformes	Clariidae	25.80	1.40	0.80	16	2.60	1.30
Clarias batrachus	Philippine Catfish	Siluriformes	Clariidae	10.93	0.80	0.53	15		
Cleisthenes pinetorum	Pointhead flounder	Pleuronectiformes	Pleuronectidae	18.10	4.00				
Clidoderma asperrimum	Roughscale sole	Pleuronectiformes	Pleuronectidae	17.40	0.60				
Clupea harengus	Atlantic Herring	Clupeiformes	Clupeidae	17.96	1.10	0.99			9.50
Clupea pallasii	Pacific herring	Clupeiformes	Clupeidae	16.85	0.90	1.10	18	17.40	22.00
Cobitis sinensis	Siberian spiny loach	Salmoniformes	Salmonidae	17.50	2.70				

Species	Common name	Order	Family	Protein	Iron	Zinc	Vit A	Vit B12	Vit D
Coilia dussumieri	Goldspotted Gredier Anchovy	Clupeiformes	Engraulidae	13.20	4.50	1.25			
Coilia nasus	Japanese gredier anchovy	Clupeiformes	Engraulidae	15.90	1.10				
Collichthys niveatus	Collichthys niveatus	Perciformes	Sciaenidae	17.00	1.30				
Cololabis saira	Pacific saury	Beloniformes	Scomberesocidae	20.15	1.50	0.80	16	15.40	14.90
Conger myriaster	Conger eel	Anguilliformes	Congridae	17.35	0.65	0.70	500	2.30	0.40
Conger orbignianus	Argentine Conger	Anguilliformes	Congridae	17.20					
Coregonus autumnalis	Arctic Cisco	Salmoniformes	Coregonidae	16.70	0.82	0.63			
Coregonus clupeaformis	Lake Whitefish	Salmoniformes	Coregonidae	16.88	0.31	0.51	11		4.40
Coreoperca kawamebari	Spotear brook perch	Perciformes	Percichthyidae	19.50	1.10				
Coryphaena hippurus	Dolphinfish	Perciformes	Coryphaenidae	17.30	0.70	0.50	8	2.60	5.00
Cottus poecilopterus	Alpine bullhead	Scorpaeniformes	Cottidae	15.00	2.80				
Cottus pollux	Japanese Sculpin/Japanese Fluvial Sculpin	Scorpaeniformes	Cottidae	15.00	2.80	1.70	180	28.20	3.00
Crossocheilus latius	Stone Roller	Cypriniformes	Cyprinidae	15.30	2.20	1.09			
Ctenopharyngodon idella	Grass carp	Cypriniformes	Cyprinidae	17.10	1.70				
Cynoglossus joyneri	Red tonguesole	Pleuronectiformes	Cynoglossidae	18.60	1.00				
Cynoglossus semilaevis	Tongue sole	Pleuronectiformes	Cynoglossidae	19.20	0.30				
Cynoscion striatus	Striped Weakfish	Perciformes	Sciaenidae	17.80	2.20				
Cyprinus carpio	Common carp	Cypriniformes	Cyprinidae	17.97	0.93	0.97	3	10.00	10.30
Cyprinus carpio nudus	Israeli carp	Cypriniformes	Cyprinidae	16.80	1.20				
Dactyloptena peterseni	Starry flying gurrd	Scorpaeniformes	Dactylopteridae	19.20	1.20				
Decapterus maruadsi	White-tipped mackerel scad	Perciformes	Cheilodactylidae	18.45	1.45	1.30	11	9.90	18.70
Decapterus muroadsi	Brownstriped Mackerel Scad	Perciformes	Carangidae	23.60	1.60	1.00	4	12.80	6.00
Dentex tumifrons	Yellow Sea Bream	Lophiiformes	Lophiidae	19.40	0.35	0.40	50	3.20	4.00
Dexistes rikuzenius	Rikuzen flounder	Pleuronectiformes	Pleuronectidae	19.40	0.60				
Diagramma pictum	Painted Sweetlips	Perciformes	Haemulidae	19.80	0.40	0.60	29	2.11	
Dictyosoma burgeri	Ribbed gunnel	Perciformes	Stichaeidae	22.00	1.20				
Dissostichus eleginoides	Patagonian Toothfish	Perciformes	Nototheniidae	13.30	0.10	0.30	1800	0.60	17.00
Ditrema temminckii temminckii	Surffish	Perciformes	Embiotocidae	18.90	1.00				
Doederleinia berycoides	Blackthroat seaperch	Perciformes	Acropomatidae	22.30	0.30				
Eleginus gracilis	Saffron cod	Gadiformes	Gadidae	12.60	4.00				
Engraulis anchoita hubbs larini	Argentine Anchovy	Clupeiformes	Engraulidae	19.20					
Engraulis encrasicolus	European Anchovy	Clupeiformes	Engraulidae	22.80	0.90	1.90	1	2.10	3.50
Engraulis japonicus	Anchovy	Clupeiformes	Engraulidae	17.95	2.25	1.00	11	13.90	4.00
Engraulis ringens	Anchoveta	Clupeiformes	Engraulidae	21.00	1.40				
Epinephelus aeneus	White Grouper	Perciformes	Serranidae	33.80	1.40	0.65	30	6.60	7.50
Epinephelus akaara	Red-spotted grouper	Perciformes	Serranidae	21.00	1.30				
Epinephelus awoara	Yellow grouper	Perciformes	Serranidae	19.40	0.90				

Species	Common name	Order	Family	Protein	Iron	Zinc	Vit A	Vit B12	Vit D
Epinephelus epistictus	Dotted grouper	Perciformes	Serranidae	19.30	1.00				
Esox lucius	Northern Pike	Esociformes	Esocidae	17.83	0.18	0.70			
Ethmalosa fimbriata	Bonga Shad	Clupeiformes	Clupeidae	19.10	1.70	1.60			
Etrumeus teres	Bigeye sardine	Clupeiformes	Clupeidae	19.90	1.90	1.30	130	14.20	9.00
Eumicrotremus orbis	Pacific spiny lumpsucker	Scorpaeniformes	Cyclopteridae	7.20	1.20				
Euthynnus affinis	Kawakawa	Perciformes	Scombridae	25.00	1.30	0.27	31		3.12
Eutropiichthys vacha	Batchwa Vacha	Siluriformes	Schilbeidae	16.10	0.70				
Evistias acutirostris	Striped boarfish	Perciformes	Pentacerotidae	18.60	1.20				
Gadus macrocephalus	Pacific cod	Gadiformes	Gadidae	18.55	0.30	0.50	10	1.30	1.00
Gadus morhua	Atlantic cod	Gadiformes	Gadidae	17.76	0.25	0.40	12	0.91	2.76
Genypterus blacodes	Pink Cusk-Eel	Ophidiiformes	Ophidiidae	15.80					
Genypterus capensis	Kingclip	Ophidiiformes	Ophidiidae	18.20	0.30	0.50	5	1.30	0.00
Girella punctata	Girella	Perciformes	Kyphosidae	19.80	0.70	0.90	55	1.80	1.00
Glossanodon semifasciatus	Deep-sea smelt	Salmoniformes	Argentinidae	18.70	0.40	0.40	75	3.40	0.00
Glossogobius giuris	Tank Goby	Perciformes	Gobiidae	14.70	1.20	0.93			
Glyptocephalus stelleri	Blackfin flounder	Pleuronectiformes	Pleuronectidae	18.20	0.60				
Gnathopogon caerulescens	Willow Shiner	Cypriniformes	Cyprinidae	17.50	1.30	3.40	250	9.00	5.00
Gudusia chapra	Indian River Shad	Clupeiformes	Clupeidae	15.40	4.80	1.97	6		
Gymnocanthus herzensteini	Black edged sculpin	Scorpaeniformes	Cottidae	19.50	2.50				
Halichoeres tenuispinis	Motley stripe rainbowfish	Perciformes	Labridae	19.20	1.10				
Halieutaea stellata	Minipizza bat fish	Lophiiformes	Ogcocephalidae	15.80	1.00				
Hapalogenys mucronatus	Broadbanded velvetchin	Perciformes	Haemulidae	18.50	1.10				
Hapalogenys nigripinnis	Short barbeled velvetchin	Perciformes	Haemulidae	19.00	1.30				
Helicolenus dactylopterus	Blackbelly Rosefish	Scorpaeniformes	Scorpaenidae	17.10					
Helicolenus hilgendorfii	Hilgendorf saucord; Rosefish	Scorpaeniformes	Scorpaenidae	16.70	0.70				
Hemibarbus labeo	Barbel steed	Cypriniformes	Cyprinidae	17.30	1.00				
Hemibarbus longirostris	Long-nose barbel	Cypriniformes	Cyprinidae	17.70	1.40				
Hemitripterus villosus	Shaggy sea raven	Scorpaeniformes	Hemitripteridae	18.60	0.50				
Henicorhynchus siamensis	Siamese Mud Carp	Cypriniformes	Cyprinidae	6.25				1.60	
Heteropneustes fossilis	Stinging Catfish	Siluriformes	Heteropneustidae	17.20	2.10	0.55	16		
Heteropriacanthus cruentatus	Glasseye	Perciformes	Priacanthidae	18.00	1.10				
Hexagrammos agrammus	Spottybelly greenling	Scorpaeniformes	Hexagrammidae	17.50	2.90				
Hexagrammos octogrammus	Masked greenling	Scorpaeniformes	Hexagrammidae	17.50	1.80				
Hexagrammos otakii	Fat greenling	Scorpaeniformes	Hexagrammidae	20.05	0.35	0.50	6	2.20	9.00
Hime japonica	Hime japonica	Aulopiformes	Aulopidae	20.90	1.00				
Hippoglossus stenolepis	Pacific Halibut	Pleuronectiformes	Pleuronectidae	19.90	0.10	0.50	13	2.10	3.00
Histiopterus typus	Sailfin armourhead	Perciformes	Pentacerotidae	19.30	1.20				
Hoplias malabaricus	Trahira	Characiformes	Erythrinidae	18.10					

Species	Common name	Order	Family	Protein	Iron	Zinc	Vit A	Vit B12	Vit D
Hoplobrotula armata	Armored weasel-fish	Ophidiiformes	Ophidiidae	18.20	1.00				
Hypomesus japonicus	Japanese Surf Smelt	Osmeriformes	Osmeridae	19.50	0.30	1.30	4	5.40	1.00
Hypomesus nipponensis	Japanese Smelt/Pond Smelt	Osmeriformes	Osmeridae	14.40	0.90	2.00	99	7.90	2.00
Hypomesus olidus	Pond smelt	Salmoniformes	Osmeridae	18.40	0.90				
Hypophthalmichthys molitrix	Silver big-head carp	Cypriniformes	Cyprinidae	18.25	1.35	0.28			
Hypophthalmichthys nobilis	Big-head carp	Cypriniformes	Cyprinidae	17.50	1.20				
Hypoptychus dybowskii	Korean sandlance	Gasterosteiformes	Hypoptychidae	21.50	1.70				
Hyporhamphus sajori	Half beak	Beloniformes	Hemiramphidae	18.70	1.90	1.90		5.50	3.00
Hyporthodus septemfasciatus	Convict grouper	Perciformes	Serranidae	18.80	1.00				
Ilisha elongata	Elongate ilisha	Clupeiformes	Pristigasteridae	20.10	0.80				
Inimicus japonicus	Devil stingfish	Scorpaeniformes	Synceiidae	19.05	0.50	0.70	2	0.60	1.00
Johnius grypotus	Corvi	Perciformes	Sciaenidae	15.20	2.60				
Kajikia audax	Barred marlin	Perciformes	Istiophoridae	23.10	0.60	0.60	8	4.30	12.00
Kareius bicoloratus	Stone flounder	Pleuronectiformes	Pleuronectidae	20.00	0.20				
Katsuwonus pelamis	Skipjack tuna	Perciformes	Scombridae	25.45	1.85	0.90	20	8.60	9.00
Konosirus punctatus	Gizzard shad	Clupeiformes	Clupeidae	19.10	1.25	0.70		10.20	9.00
Labeo bata	Bata	Cypriniformes	Cyprinidae	15.90	1.20	0.94			
Labeo calbasu	Orangefin Labeo	Cypriniformes	Cyprinidae	17.00	1.10	0.36			
Labeo gonius	Kuria Labeo	Cypriniformes	Cyprinidae	17.60	0.30	0.06			
Labeo rohita	Roho Labeo	Cypriniformes	Cyprinidae	20.60	0.40	1.13	4		
Labracoglossa argenteiventris	Yellowstriped butterfish	Perciformes	Kyphosidae	18.70	0.60	1.30	16	2.00	4.00
Lagocephalus lunaris	Lurtail puffer	Tetraodontiformes	Tetraodontidae	17.30	1.00				
Larimichthys crocea	Large yellow croaker	Perciformes	Sciaenidae	17.20	1.20				
Larimichthys polyactis	Yellow croaker	Perciformes	Sciaenidae	15.80	0.50				
Lateolabrax japonicus	Common sea bass	Perciformes	Percichthyidae	18.83	0.80	0.50	180	2.00	10.00
Lates calcarifer	Barramundi	Perciformes	Centropomidae	18.60	1.00	0.16	8		1.50
Lepidopsetta mochigarei	Dusky sole	Pleuronectiformes	Pleuronectidae	16.80	1.20				
Lepidotrigla alata	Fork-snout searobin	Scorpaeniformes	Triglidae	16.90	1.00				
Lepidotrigla microptera	Red-wing searobin	Scorpaeniformes	Triglidae	19.70	0.40				
Lepomis macrochirus	Bluegill	Perciformes	Centrarchidae	17.80	1.40				
Leptomelanosoma indicum	Indian Threadfin	Perciformes	Polynemidae	20.30	0.50	1.35			
Lethrinus haematopterus	Chinese emperor	Perciformes	Lethrinidae	20.40	1.00				
Lethrinus nebulosus	Spangled Emperor	Perciformes	Lethrinidae	20.50	0.30	0.50	8	3.70	11.00
Limanda aspera	Yellowfin sole	Pleuronectiformes	Pleuronectidae	16.80	1.20				
Liparis tanakai	Taka's silfish	Scorpaeniformes	Liparidae	16.40	0.50				
Liparis tessellatus	Cubed silfish	Scorpaeniformes	Liparidae	16.40	1.00				
Lobotes surinamensis	Tripletail	Perciformes	Lobotidae	21.00	1.10				
Lophiomus setigerus	Angler	Lophiiformes	Lophiidae	14.10	2.50				

Species	Common name	Order	Family	Protein	Iron	Zinc	Vit A	Vit B12	Vit D
Lophius litulon	Yellow goosefish	Perciformes	Sciaenidae	14.65	0.25	0.60	13	1.20	1.00
Lota lota	Burbot	Gadiformes	Lotidae	17.83	0.35	0.65	3		
Luciopimelodus pati	Pati	Siluriformes	Pimelodidae	18.20	1.90				
Lumpenus sagitta	Ske prickleback	Perciformes	Stichaeidae	19.30	0.90				
Macruronus novaezelandiae	Hoki	Gadiformes	Merlucciidae	17.00	0.30	0.40	43	0.70	1.00
Makaira mazara	Pacific Blue Marlin/Blue Marlin/Black Marlin	Perciformes	Istiophoridae	22.90	0.50	0.70	2	1.50	38.00
Malakichthys wakiyae	Silver-belly sea perch	Perciformes	Acropomatidae	18.70	0.70				
Mallotus villosus	Capelin	Salmoniformes	Osmeridae						
Mastocembellus armatus	Zig-Zag Eel	Synbranchiformes	Mastacembelidae	16.10	0.80	1.30	829	2.40	
Merluccius merluccius	European Hake	Gadiformes	Merlucciidae	17.10	1.90				
Microcanthus strigatus	Stripey	Perciformes	Kyphosidae	19.50	0.90				
Micromesistius australis	Southern Blue Whiting	Gadiformes	Gadidae	16.40	0.30	0.30	6	1.60	7.00
Micromesistius poutassou	Blue Whiting	Gadiformes	Gadidae	17.50	1.00				
Micropogonias undulatus	Atlantic Croaker	Perciformes	Sciaenidae	19.50					
Micropterus salmoides	Largemouth black bass	Perciformes	Centrarchidae	18.20	4.50				
Miichthys miiuy	Mi-iuy croaker	Perciformes	Sciaenidae	18.00	0.30				
Misgurnus anguillicaudatus	Loach	Cypriniformes	Cobitidae	16.15	6.80	2.90	15	8.50	4.00
Monopterus albus	Asian swamp eel	Synbranchiformes	Synbranchidae	17.90	1.40				
Mugil cephalus	Common mullet	Mugiliformes	Mugilidae	20.45	0.85	0.50	8	4.70	10.00
Muraenesox cinereus	Silver conger eel	Anguilliformes	Muraenesocidae	20.10	1.15	0.60	59	1.90	5.00
Mystus cavasius	Gangetic Mystus	Siluriformes	Bagridae	15.40	1.30	0.88			
Mystus gulio	Long Whiskers Catfish	Siluriformes	Bagridae	17.00	0.90	0.23			
Mystus wolffi	Mystus wolfii	Siluriformes	Bagridae	6.25					
Nandus nandus	Gangetic Leaffish	Perciformes	ndidae	15.80	1.90	1.42			
Nemadactylus bergi	Castaneda	Perciformes	Cheilodactylidae	18.40					
Nemipterus japonicus	Japanese Threadfin Bream	Perciformes	Nemipteridae	18.40	0.80	0.30	0	2.00	
Nemipterus virgatus	Golden threadfin bream	Perciformes	Nemipteridae	19.80	0.70				
Neoditrema ransonnetii	Surfperch	Perciformes	Embiotocidae	21.80	1.80				
Niphon spinosus	Ara	Perciformes	Serranidae	19.30	1.40				
Notopterus notopterus	Bronze Featherback	Osteoglossiformes	Notopteridae	17.80	1.00	0.74	30		
Odentesthes incisa	Silverside	Atheriniformes	Atherinidae	16.00	2.00				
Odontesthes bonariensis	Argentinian Silverside	Atheriniformes	Atherinopsidae	18.30	1.90				
Odontesthes regia	Chilean Silverside	Atheriniformes	Atherinopsidae	18.60					
Odontobutis platycephala	Fresh-water goby	Gobioidei	Odontobutidae	16.90	1.00				
Ompok pabda	Pabdah Catfish	Siluriformes	Siluridae	17.30	1.20	1.25			
Oncorhynchus gorbuscha	Pink Salmon	Salmoniformes	Salmonidae	21.70	0.40	0.60	13	4.60	22.00
Oncorhynchus keta	Chum salmon	Salmoniformes	Salmonidae	21.45	0.80	0.50	11	5.90	32.00
Oncorhynchus kisutch	Coho salmon	Salmoniformes	Salmonidae	20.65	1.40	0.60	36	5.20	15.00

Species	Common name	Order	Family	Protein	Iron	Zinc	Vit A	Vit B12	Vit D
Oncorhynchus masou	Cherry salmon	Salmoniformes	Salmonidae	20.60	1.20	0.50	63	7.60	10.00
Oncorhynchus masou ishikawae	Masu Salmon	Salmoniformes	Salmonidae	18.30	0.40	0.80	7	5.50	9.00
Oncorhynchus mykiss	Rainbow trout	Salmoniformes	Salmonidae	21.10	1.80				
Oncorhynchus nerka	Sockeye salmon	Salmoniformes	Salmonidae	22.50	0.40	0.50	27	9.40	33.00
Oncorhynchus tshawytscha	Chinook Salmon	Salmoniformes	Salmonidae	18.80	0.25	0.35	111	2.35	16.00
Oplegnathus fasciatus	Rock bream	Perciformes	Oplegthidae	18.65	0.45	0.60	39	1.30	3.00
Oplegnathus punctatus	Spotted knifejaw	Perciformes	Oplegthidae	18.90	0.70				
Oreochromis mossambicus	Mozambique tilapia	Perciformes	Cichlidae	20.05	0.80	1.40	2		5.50
Oreochromis niloticus	Nile Tilapia	Perciformes	Cichlidae	19.80	0.50	0.40	3	2.30	11.00
Osmerus mordax	Pacific rainbow smelt	Salmoniformes	Osmeridae	17.40	1.40				
Ostichthys japonicus	Big-eye soldierfish	Beryciformes	Holocentridae	21.30	1.20				
Pagrus major	Genuine porgy	Perciformes	Sparidae	19.50	0.35	0.40	8	1.20	5.00
Pagrus pagrus	Red Porgy	Perciformes	Sparidae	20.40	1.80	3.30			
Pampus argenteus	Silver/white pomfret	Perciformes	Stromateidae	17.13	0.40	0.39	90	1.40	5.00
Pampus chinensis	Chinese Silver Pomfret	Perciformes	Stromateidae	15.90	0.40	0.59			
Pangasianodon hypophthalmus	Striped Catfish	Siluriformes	Pangasiidae	6.25				4.50	
Pangasius pangasius	Pangas Catfish	Siluriformes	Pangasiidae	15.90	0.10	1.85	5		
Parabembras curtus	Matron flathead	Scorpaeniformes	Bembridae	20.10	1.30				
Parajulis poecilopterus	Multicolor finfish	Perciformes	Labridae	19.30	2.60				
Paralichthys olivaceus	Bastard halibut	Pleuronectiformes	Paralichthyidae	20.75	0.38	0.40	12	1.00	3.00
Parapercis multifasciata	Bicolor-barred weever	Perciformes	Pinguipedidae	18.30	0.90				
Parapercis sexfasciata	Grub fish	Perciformes	Pinguipedidae	20.80	1.30				
Parapristipoma trilineatum	Grunt	Perciformes	Haemulidae	17.10	0.80	0.60	41	5.80	15.00
Parascolopsis inermis	Unarmed dwarf monocle bream	Perciformes	Nemipteridae	19.20	0.60				
Parastromateus niger	Black Pomfret	Perciformes	Carangidae	19.60	0.90	0.48			
Parona signata	Paro Leatherjacket	Perciformes	Carangidae	20.10					
Pennahia argentata	White croaker	Perciformes	Sciaenidae	18.65	0.40	0.60	5	2.50	2.90
Percophis brasiliensis	Brazilian Flathead	Perciformes	Percophidae	20.10					
Pholis nebulosa	Tidepool gunnel	Perciformes	Pholidae	20.70	1.10				
Platycephalus indicus	Bartailed flathead	Scorpaeniformes	Platycephalidae	20.00	0.60				
Plecoglossus altivelis	Sweet fish	Salmoniformes	Plecoglossidae	17.50	1.10	0.80	35	10.30	1.00
Plectorhynchus cinctus	Crescent sweetlips	Perciformes	Haemulidae	19.50	0.70				
Pleurogrammus azonus	Arabesque greenling	Scorpaeniformes	Hexagrammidae	18.45	4.15	1.10	25	10.70	3.00
Pleuronectes herzensteini	Brown sole	Pleuronectiformes	Pleuronectidae	19.50	0.35	0.80	5	3.10	13.00
Pleuronectes yokohamae	Marbled sole	Pleuronectiformes	Pleuronectidae	18.95	0.35	0.80	6	1.80	6.70
Pleuronichthys cornutus	Ridged-eye flounder	Pleuronectiformes	Pleuronectidae	17.60	1.10				
Polynemus quadrifilis	Giant African Threadfin	Perciformes	Polynemidae	17.60	0.30	1.00			
Pomatomus saltatrix	Bluefish	Perciformes	Pomatomidae	21.50	1.40				

Species	Common name	Order	Family	Protein	Iron	Zinc	Vit A	Vit B12	Vit D
Priacanthus macracanthus	Red bigeye	Perciformes	Priacanthidae	18.90	1.20				
Prionotus punctatus	Bluewing Searobin	Scorpaeniformes	Triglidae	18.90					
Protonibea diacanthus	Blacksotted Croaker	Perciformes	Sciaenidae	18.60	0.40	0.65	17		0.60
Psenopsis anomala	Butterfish	Perciformes	Centrolophidae	16.35	0.55	0.80	95	2.70	2.00
Pseudaesopia japonica	Wavy-banded sole	Pleuronectiformes	Soleidae	18.40	1.10				
Pseudambassis ranga	Indian Glassy Catfish	Perciformes	Ambassidae	15.50	2.00	2.45	106		
Pseudoblennius cottoides	Sunrise sculpin	Scorpaeniformes	Cottidae	22.40	1.70				
Pseudocaranx dentex	Striped Jack/White Trevally	Perciformes	Carangidae	21.90	0.70	1.10	10	3.20	18.00
Pseudogobio esocinus	Pike gudgeon	Cypriniformes	Cyprinidae	15.70	1.20				
Pseudolabrus japonicus	Bambooleaf wrasse	Perciformes	Labridae	18.90	1.10				
Pseudoplatystoma corruscans	Spotted Sorubim	Siluriformes	Pimelodidae	18.20	1.90				
Pseudorasbora parva	Stone moroko	Cypriniformes	Cyprinidae	16.40	2.50				
Pterocaesio digramma	Double-Lined Fusilier	Perciformes	Caesionidae	20.20	0.50	0.70	7	4.40	2.00
Puntius sophore	Pool Barb	Cypriniformes	Cyprinidae	17.60	2.00	3.00			
Rastrelliger brachysoma	Short Mackerel	Perciformes	Scombridae	6.25					
Rastrelliger kanagurta	Indian mackerel	Perciformes	Scombridae	20.51					
Rexea prometheoides	Royal escolar	Perciformes	Gempylidae	20.00	0.80				
Salangichthys microdon	Icefish	Salmoniformes	Salangidae	13.45	0.55	1.20	50	3.30	1.00
Salminus maxillosus	Dorado	Characiformes	Bryconidae	18.80					
Salmo salar	Atlantic Salmon	Salmoniformes	Salmonidae	20.10	0.30	0.40	17	8.90	10.00
Salmophasia bacaila	Large Razorbelly Minnow	Cypriniformes	Cyprinidae	18.10	5.40	3.10			
Salmophasia phulo	Finescale Razorbelly Minnow	Cypriniformes	Cyprinidae	15.30	1.90	3.10			
Salvelinus alpinus	Arctic Char	Salmoniformes	Salmonidae	18.75	0.34	0.42	32		25.80
Salvelinus leucomaenis	White-Spotted Char/Char/Japanese Char	Salmoniformes	Salmonidae	19.00	0.30	0.80	5	4.20	5.00
Salvelinus namaycush	Lake Trout	Salmoniformes	Salmonidae	15.78	0.23	0.62	40		19.70
Sarda orientalis	Striped bonito	Perciformes	Scombridae	24.60	2.10				
Sarda sarda	Atlantic Bonito	Perciformes	Scombridae	22.00					
Sardina pilchardus	European pilchard	Clupeiformes	Clupeidae	17.33					21.20
Sardinella zunasi	Japanese sardinella	Clupeiformes	Clupeidae	16.30	1.90				
Sardinops sagax	South American Pilchard	Clupeiformes	Clupeidae	18.77	2.00	1.60	8	15.70	32.00
Satyrichthys rieffeli	Spotted armored-gurrd	Scorpaeniformes	Peristediidae	19.30	2.10				
Saurida undosquamis	Lizard fish	Aulopiformes	Synodontidae	20.80	0.40				
Scomber australasicus	Blue Mackerel	Perciformes	Scombridae	23.00	1.60	1.10	8	12.60	4.30
Scomber colias	Black Drum	Perciformes	Sciaenidae	18.80					
Scomber japonicus	Mackerel	Perciformes	Scombridae	19.00	1.60	1.10	37	12.90	5.10
Scomber scombrus	Atlantic Mackerel	Perciformes	Scombridae	17.20	0.90	0.90	44	8.10	10.00
Scomberomorus cavalla	King Mackerel	Perciformes	Scombridae	20.90	0.50	0.60	17	0.55	

Species	Common name	Order	Family	Protein	Iron	Zinc	Vit A	Vit B12	Vit D
Scomberomorus commerson	rrow-Barred Spanish Mackerel	Perciformes	Scombridae	19.80	2.00	0.40	30		
Scomberomorus guttatus	Indo-Pacific King Mackerel	Perciformes	Scombridae	20.90	0.80	0.78			
Scomberomorus koreanus	Korean seerfish	Perciformes	Scombridae	17.90	0.70				
Scomberomorus niphonius	Spanish mackerel	Perciformes	Scombridae	19.73	0.70	0.80	12	4.00	7.00
Scombrops boops	Japanese blue fish	Perciformes	Scombropidae	18.75	0.65	0.40	8	1.90	4.00
Scorpaena neglecta	Izu scorpionfish sting fish	Scorpaeniformes	Scorpaenidae	15.50	0.90				
Scorpaenopsis cirrhosa	Weedy stingfish	Scorpaeniformes	Scorpaenidae	19.00	1.10				
Sebastes alutus	Pacific Ocean perch, Longjaw rockfish	Scorpaeniformes	Scorpaenidae	16.45	0.25	0.40	20	1.60	3.00
Sebastes hubbsi	Amorclad rockfish	Scorpaeniformes	Scorpaenidae	18.30	0.90				
Sebastes inermis	Black rock fish	Scorpaeniformes	Scorpaenidae	19.30	0.60				
Sebastes iracundus	Angry Rockfish	Scorpaeniformes	Scorpaenidae	16.30	0.20	0.40	85	3.30	3.00
Sebastes matsubarae	Matsubara's Red Rockfish	Scorpaeniformes	Scorpaenidae	16.80	0.30	0.40	26	0.70	1.00
Sebastes oblongus	Oblong rockfish	Scorpaeniformes	Scorpaenidae	21.30	0.70				
Sebastes pachycephalus	Spotbelly rockfish	Salmoniformes	Salmonidae	19.10	0.80				
Sebastes schlegelii	Korean rockfish	Scorpaeniformes	Scorpaenidae	21.40	0.50				
Sebastes taczanowskii	White-edged rockfish	Perciformes	Sciaenidae	19.70	1.70				
Sebastes thompsoni	Gold eye rockfish	Scorpaeniformes	Scorpaenidae	18.60	1.50				
Sebastes vulpes	Fox jacopever	Scorpaeniformes	Scorpaenidae	22.40	1.00				
Sebastiscus marmoratus	Scorpion fish	Scorpaeniformes	Scorpaenidae	19.60	0.45	0.50	3	1.20	2.00
Sebastolobus macrochir	Kichiji Rockfish/ Broadbanded Thornyhead	Scorpaeniformes	Sebastidae	13.60	0.30	0.40	65	1.00	4.00
Semicossyphus reticulatus	Cold porgy	Perciformes	Labridae	18.50	0.30				
Seriola dumerili	Greater Amberjack	Perciformes	Carangidae	21.00	0.60	0.70	4	5.30	4.00
Seriola lalandi	Amberjack	Perciformes	Carangidae	22.45	0.85	0.70	19	2.10	5.00
Seriola quinqueradiata	Yellow tail	Perciformes	Carangidae	21.20	1.00	0.70	50	3.80	8.00
Seriolella punctata	Silver Warehou	Perciformes	Centrolophidae	18.60	0.60	0.50	100	1.80	3.00
Setipinna phasa	Gangetic Hairfin Anchovy	Clupeiformes	Engraulidae	17.70	1.80	3.20	12		
Setipinna taty	Scaly Hairfin Anchovy	Clupeiformes	Engraulidae	19.30	2.30	1.60	8		
Siganus fuscescens	Mottled spinefooot	Perciformes	Siganidae	20.30	1.20				
Sillago japonica	Japanese Whiting	Perciformes	Sillaginidae	18.50	0.10	0.40	1	2.20	0.70
Sillago sihama	Silver sillago	Perciformes	Sillaginidae	19.50	2.30				
Silurus asotus	Amur catfish	Siluriformes	Siluridae	15.10	0.80				
Siniperca scherzeri	Leopard mandarin fish	Perciformes	Percichthyidae	17.20	2.10				
Sperata seenghala	Giant River-Catfish	Siluriformes	Bagridae	15.90	0.70	0.23			
Sphyraena japonica	Japanese barracuda	Perciformes	Sphyraenidae	18.90	0.30				
Sphyraena pinguis	Red barracuda	Perciformes	Sphyraenidae	19.70	0.60	0.50	12	2.30	11.00
Spratelloides gracilis	Blue Sprat	Clupeiformes	Clupeidae	18.80	1.10	1.90		8.30	10.00
Sprattus fuegensis	Falkland Sprat	Clupeiformes	Clupeidae	14.00					

Species	Common name	Order	Family	Protein	Iron	Zinc	Vit A	Vit B12	Vit D
Sprattus sprattus	Sprat	Clupeiformes	Clupeidae	22.63					
Stenodus leucichthys	Sheefish	Salmoniformes	Salmonidae	19.00	0.40	0.30			
Stephanolepis cirrhifer	Thread-sail filefish	Tetraodontiformes	Mocanthidae	18.95	0.30	0.40	2	1.30	43.00
Stichaeus grigorjewi	Long shanny	Perciformes	Stichaeidae	19.10	1.20				
Strangomera bentincki	Araucanian herring	Clupeiformes	Clupeidae	16.50					
Suggrundus meerdervoorti	Big-eyed flathead	Scorpaeniformes	Platycephalidae	17.50	0.20	0.60	2	3.00	11.00
Synchiropus altivelis	Highfin bigeye dragonet	Perciformes	Callionymidae	16.90	0.60				
Synodus myops	Snakefish	Aulopiformes	Synodontidae	20.00	0.60				
Systomus sarana	Olive Barb	Cypriniformes	Cyprinidae	17.40	0.60	0.74			
Tachysurus fulvidraco	Yellow catfish	Siluriformes	Bagridae	15.55	0.90				
Takifugu pardalis	Panther puffer	Tetraodontiformes	Tetraodontidae	18.90	2.50				
Takifugu poecilonotus	Fine-patterned puffer	Tetraodontiformes	Tetraodontidae	15.90	1.00				
Takifugu porphyreus	Purple puffer	Tetraodontiformes	Tetraodontidae	20.20	0.25	1.50	7	3.00	6.00
Takifugu rubripes	Tiger puffer	Tetraodontiformes	Tetraodontidae	19.05	0.25	0.90	3	1.90	4.00
Takifugu stictonotus	Spotty-back puffer	Tetraodontiformes	Tetraodontidae	19.30	3.10				
Takifugu vermicularis	Purple puffer	Tetraodontiformes	Tetraodontidae	20.25	1.20				
Takifugu xanthopterus	Yellowfin puffer	Tetraodontiformes	Tetraodontidae	19.30	4.40				
Tanakius kitaharae	Willowy flounder	Perciformes	Carangidae	18.30	1.90				
Tenualosa ilisha	Hilsha Shad	Clupeiformes	Clupeidae	18.00	1.30	0.54			
Thamnaconus modestus	Black scraper	Tetraodontiformes	Mocanthidae	18.05	0.45	0.50		1.40	8.00
Theragra chalcogramma	Alaska pollack	Gadiformes	Gadidae	17.45	0.85	0.50	10	2.90	0.50
Thunnus alalunga	Albacore	Perciformes	Scombridae	26.00	0.90	0.50	4	2.80	7.00
Thunnus albacares	Yellowfin tuna	Perciformes	Scombridae	24.10	1.80	0.50	2	5.80	6.00
Thunnus maccoyii	Southern Bluefin Tuna	Perciformes	Scombridae	23.30	1.60	0.50	39	0.70	
Thunnus obesus	Big-Eye Tuna	Perciformes	Scombridae	22.80	1.40	0.40	3	4.50	2.00
Thunnus thynnus	Bluefin tuna	Perciformes	Scombridae	26.20	2.05	0.50	61	6.90	12.00
Thymallus arcticus	Arctic Grayling	Salmoniformes	Salmonidae	20.00	1.00	0.70			
Thyrsites atun	Barracouta	Perciformes	Gempylidae	21.70	0.60	0.50	55	6.50	2.00
Trachipterus ishikawae	Slender ribbonfish	Lampriformes	Trachipteridae	18.10	0.30				
Trachurus japonicus	Horse mackerel	Perciformes	Carangidae	20.20	0.75	1.10	7	7.10	8.90
Trachurus picturatus	Bluejack Mackerel	Perciformes	Carangidae	19.00					
Trachurus trachurus	Atlantic Horse Mackerel	Perciformes	Carangidae	19.60	1.00	0.90	16	8.10	8.00
Tribolodon hakonensis	Far eastern dace	Cypriniformes	Cyprinidae	19.20	1.10	3.40	41	8.50	19.00
Trichiurus lepturus	Hair tail	Perciformes	Trichiuridae	17.50	0.60	0.50	52	0.90	14.00
Trichogaster fasciata	Banded Gourami	Perciformes	Belontiidae	15.80	0.90	1.35	39		
Umbrina canosai	Argentine Croaker	Perciformes	Sciaenidae	19.10					
Upeneus japonicus	Bensasi goatfish	Perciformes	Mullidae	7.10	1.40				
Uranoscopus japonicus	Stargazer	Perciformes	Uranoscopidae	18.20	0.70				
Urophycis brasiliensis	Brazilian Codling	Gadiformes	Phycidae	17.60	2.10				

Species	Common name	Order	Family	Protein	Iron	Zinc	Vit A	Vit B12	Vit D
Verasper variegatus	Spotted halibut	Pleuronectiformes	Pleuronectidae	18.20	0.20				
Wallago attu	Wallago	Siluriformes	Siluridae	15.40	0.80	0.27	1		
Xenentodon cancila	Freshwater Garfish	Beloniformes	Belonidae	16.60	0.80	0.94	65		
Xenocephalus elongatus	Blue-spotted stargazer	Perciformes	Uranoscopidae	17.40	0.40				
Xiphias gladius	Swordfish	Perciformes	Xiphiidae	18.10	3.35	0.70	61	1.90	8.80
Zacco platypus	Common minnow	Cypriniformes	Cyprinidae	18.85	1.25	2.50	10	11.30	10.00
Zalanthias kelloggi	Eastern flower porgy	Perciformes	Serranidae	19.00	1.00				
Zebrias zebra	Zebra sole	Pleuronectiformes	Soleidae	18.90	1.20				
Zenopsis nebulosa	Mirror dory	Zeiformes	Zeidae	20.30	0.80				
Zeus faber	John dory	Zeiformes	Zeidae	19.10	0.60				
Zoarces gillii	Blotched eelpout	Perciformes	Zoarcidae	18.50	0.40				

Supplementary Table 2. Nutrient content of fish species in dataset.[2,3,4,5,6,7,8,9,10,11,12,13,14,15]

Category	Nutrient	# Valid Species	Min	Max	Median	Mean	SD
Protein, fats (g/100g)	Protein	371	6.3	33.8	18.6	18.5	2.9
	Total fat	367	0.1	26.8	2.3	3.6	4.1
	Omega-3 fatty acids	238	0.01	7.68	0.69	0.97	1.12
	Omega-6 fatty acids	238	0.00	4.41	0.11	0.20	0.41
Minerals (mg/100g)	Iron	344	0.10	9.10	1.00	1.19	1.04
	Zinc	177	0.06	3.90	0.65	0.91	0.73
Vitamins (µg/100g)	Vitamin A (retinol activity equivalents)	146	0.0	2680.0	15.0	96.6	356.5
	Vitamin B12	124	0.0	28.2	2.9	4.6	4.4
	Vitamin D	122	0.0	43.0	5.0	8.1	8.2

Supplementary Table 3. Summary statistics for life history traits.

Continuous Variables	# Valid Species	Min	Max	Median	Mean	SD
**Minimum depth (m)**	259	0	320	3.0	28.15	59.4
**Maximum depth (m)**	249	2	3850	200.0	247.5	484.8
**Maximum length (cm)**	370	8.0	505.1	48.0	71.7	70.4
***a* (L/W scalar)**	333	.0004	.0263	0.0089	0.0097	0.0055
***b* (L/W exponent)**	332	2.79	3.39	3.05	3.05	0.08
**Trophic level**	373	2.0	4.7	3.6	3.6	0.5

Categorical Variables	Categories	# Species	Percent
**Habitat**	Bathydemersal	15	4.0
	Benthopelagic	90	23.9
	Demersal	149	39.6
	Pelagic	25	6.6
	Pelagic-neritic	51	13.6
	Reef-associated	44	11.7
**Latitudinal range**	Tropical	126	33.5
	Subtropical	104	27.7
	Temperate	126	33.5
	Boreal/austral	5	1.3
	Polar	8	2.1

*Supplementary Table 4. Evolutionary correlation matrix, <u>with omega-3 and omega-6 fatty acids included</u>. Correlations with p<0.1 are in **bold**.*

	**depmin**	**depmax**	**maxlen**	**a_lw**	**b_lw**	**troph**	**protein**	**fat**	**iron**	**zinc**	**vitA**	**vitb12**	**vitd**
**depmin**	1.00	**0.22**	0.07	-0.06	0.05	0.09	-0.02	0.06	-0.04	-0.08	0.17	0.00	-0.11
**depmax**	0.22	1.00	**0.46**	**-0.32**	0.15	**0.43**	-0.23	**0.37**	-0.07	-0.25	**0.46**	-0.09	0.04
**maxlen**	0.07	**0.46**	1.00	-0.13	-0.10	**0.44**	0.11	0.12	-0.06	**-0.45**	**0.31**	**-0.21**	0.13
**a_lw**	-0.06	**-0.32**	-0.13	1.00	**-0.60**	-0.22	0.13	-0.18	-0.13	-0.11	-0.13	-0.15	0.09
**b_lw**	0.05	0.15	-0.10	**-0.60**	1.00	0.00	-0.15	0.10	0.20	0.23	0.15	0.06	0.04
**troph**	0.09	**0.43**	**0.44**	-0.22	0.00	1.00	0.17	0.01	-0.13	-0.24	0.06	-0.16	-0.06
**protein**	-0.02	-0.23	0.11	0.13	-0.15	0.17	1.00	**-0.39**	0.08	0.07	**-0.41**	0.14	0.01
**lipid**	0.06	**0.37**	0.12	-0.18	0.10	0.01	**-0.39**	1.00	0.16	-0.04	**0.69**	**0.31**	**0.42**
**iron**	-0.04	-0.07	-0.06	-0.13	0.20	-0.13	0.08	0.16	1.00	**0.55**	0.21	**0.56**	**0.26**
**zinc**	-0.08	-0.25	**-0.45**	-0.11	0.23	-0.24	0.07	-0.04	**0.55**	1.00	-0.03	**0.38**	0.18
**ret_eq**	0.17	**0.46**	**0.31**	-0.13	0.15	0.06	**-0.41**	**0.69**	0.21	-0.03	1.00	0.05	**0.27**
**vitb12**	0.00	-0.09	-0.21	-0.15	0.06	-0.16	0.14	**0.31**	**0.56**	**0.38**	0.05	1.00	**0.22**
**vitd**	-0.11	0.04	0.13	0.09	0.04	-0.06	0.01	**0.42**	**0.26**	0.18	**0.27**	0.22	1.00

P-VALUES

	**depmin**	**depmax**	**maxlen**	**a_lw**	**b_lw**	**troph**	**protein**	**fat**	**iron**	**zinc**	**vitA**	**vitb12**	**vitd**
**depmin**	NA	0.085	0.340	0.351	0.372	0.301	0.392	0.351	0.376	0.322	0.157	0.397	0.271
**depmax**	0.085	NA	**0.000**	**0.014**	0.186	**0.001**	0.071	**0.004**	0.337	0.050	**0.000**	0.308	0.381
**maxlen**	0.340	**0.000**	NA	0.232	0.281	**0.000**	0.269	0.258	0.355	**0.000**	**0.016**	**0.097**	0.223
**a_lw**	0.351	**0.014**	0.232	NA	**0.000**	0.087	0.234	0.135	0.228	0.267	0.226	0.188	0.308
**b_lw**	0.372	0.186	0.281	**0.000**	NA	0.397	0.198	0.283	0.112	0.068	0.199	0.351	0.379
**troph**	0.301	**0.001**	**0.000**	0.087	0.397	NA	0.150	0.396	0.240	0.064	0.354	0.175	0.359
**protein**	0.392	0.071	0.269	0.234	0.198	0.150	NA	**0.002**	0.317	0.333	**0.001**	0.207	0.395
**lipid**	0.351	**0.004**	0.258	0.135	0.283	0.396	**0.002**	NA	0.170	0.374	**0.000**	**0.017**	**0.001**
**iron**	0.376	0.337	0.355	0.228	0.112	0.240	0.317	0.170	NA	**0.000**	0.097	**0.000**	**0.041**
**zinc**	0.322	0.050	**0.000**	0.267	0.068	0.064	0.333	0.374	**0.000**	NA	0.385	**0.003**	0.134
**ret_eq**	0.157	**0.000**	**0.016**	0.226	0.199	0.354	**0.001**	**0.000**	0.097	0.385	NA	0.362	**0.034**
**vitb12**	0.397	0.308	0.097	0.188	0.351	0.175	0.207	**0.017**	**0.000**	**0.003**	0.362	NA	0.082
**vitd**	0.271	0.381	0.223	0.308	0.379	0.359	0.395	**0.001**	**0.041**	0.134	**0.034**	0.082	NA

Supplementary Figures

Supplementary Figure 1. Residual plots for nutrient prediction: protein (A), total fat (B), iron (C), zinc (D), vitamin A (E), vitamin B12 (F), and vitamin D (G).

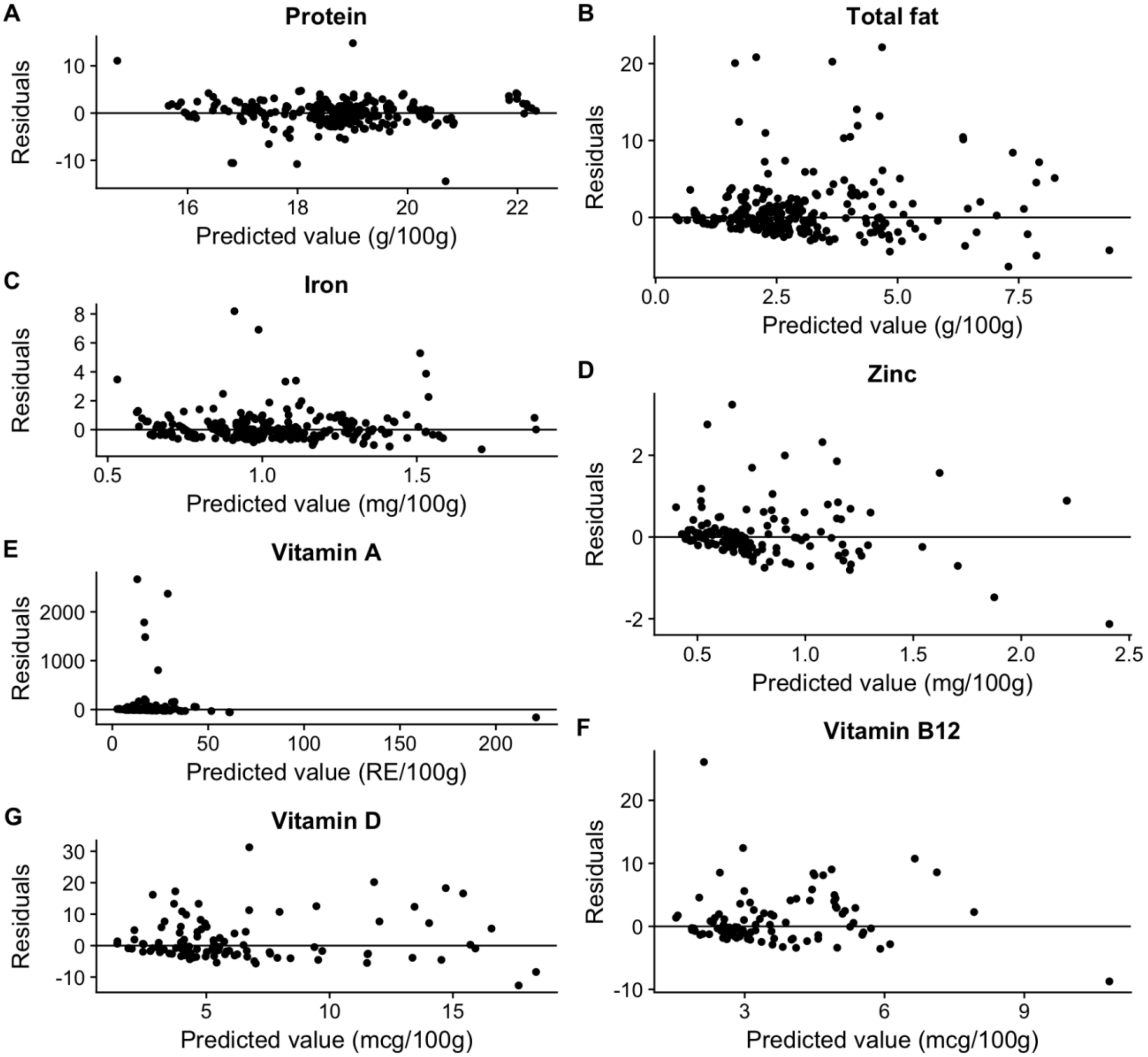

Supplementary References

[1] Food and Agricultural Organization (FAO) of the United Nations. Codex Alimentarius: Food Labeling Complete Texts. FAO/WHO (2001).

[2] Universidad Nacional de Luján. *Tabla de composicion de alimentos*. 2010. Accessed 25 July 2017 from: http://www.unlu.edu.ar/~argenfood/Tablas/Tabla.htm.

[3] Shaheen, N. et al. *Food Composition Tables for Bangladesh*. University of Dhaka (2013).

[4] Seyha, S. & Kuong, K. *Food composition table for Cambodia*. Ministry of Agriculture, Forestry and Fisheries of Cambodia (2013).

[5] Centre for Indigenous Peoples' Nutrition and Environment (McGill University). *Traditional Food Composition Nutribase*. 2005. Accessed 25 July 2017 from: https://www.mcgill.ca/cine/files/cine/Traditional_Food_Composition_Nutribase.pdf

[6] Rittenschober, D., Stadlmayr, B., Nowak, V., Du, J. and Charrondiere, U.R. Report on the development of the FAO/INFOODS user database for fish and shellfish (uFiSh) - Challenges and possible solutions. *Food Chemistry* **193**, 112–120 (2016).

[7] Prynne, C.J. & Paul, A.A. *Food Composition Table for use in The Gambia*. MRC Human Nutrition Research, Cambridge, UK (2011).

[8] Office for Resources, Policy Division Science and Technology Policy Bureau, Japan. *Standard Tables of Food Composition in Japan, Seventh Revised Edition*. 2015. Accessed 25 July 2017 from: http://www.mext.go.jp/en/policy/science_technology/policy/title01/detail01/1374030.htm

[9] Kim, J.C., Cho, Y.S., Kim, S.N., Choi, Y., Kim, J.B., Park, H.J., & Kim, H.R. *8th revision Standard Food Composition Table*. Department of Agrofood Resources, NAAS, RDA, Korea (2013).

[10] Korkalo, L., Hauta-Alus, H. and Mutanen, M. *Food composition tables for Mozambique, Version 2*. Department of Food and Environmental Sciences. University of Helsinki, Finland (2011).

[11] Sivakumaran, S., Martell, S. and Huffman, L. *The Concise New Zealand Food Composition Tables, 9th Edition*. The New Zealand Institute for Plant & Food Research Limited and Ministry of Health (2012).

[12] US Department of Agriculture (USDA), Agricultural Research Service, Nutrient Data Laboratory. *USDA National Nutrient Database for Standard Reference, Release 28 (Slightly revised)*. Accessed 25 July 2017 from: http://www.ars.usda.gov/ba/bhnrc/ndl (2016).

[13] Stadlmayr, B. et al. *Table de composition des aliments d'Afrique de l'Ouest*. Food and Agriculture Organization of the United Nations. Accessed 25 July 2017 from: http://www.fao.org/docrep/015/i2698b/i2698b00.pdf (2012).

[14] Dignan, C., Burlingame, B., Kumar, S., and Aalbersberg, W. *The Pacific Islands food composition tables, Second edition*. Food and Agriculture Organization of the United Nations. Accessed 25 July 2017 from: http://www.fao.org/docrep/007/y5432e/y5432e00.htm (2004).

[15] Rittenschober D., Nowak, V., and Charrondiere, U.R. Review of availability of food composition data for fish and shellfish. *Food Chemistry* **141**, 4304-4310 (2013).
