# Supplementary material for: Predicting nutrient content of ray-finned fishes using phylogenetic information
Source: Nat Commun. 2018 Sep 25;9:3742. doi: 10.1038/s41467-018-06199-w (PMC6156416; doi:10.1038/s41467-018-06199-w)
Supplement: Supplementary file 3 — Description of Additional Supplementary Files [file 41467_2018_6199_MOESM3_ESM.pdf]

Description of Additional Supplementary Files:

- **Supplementary data file 1**. *SD1_fish _database.csv*. Database of life history and nutrient content values for all included species.
- **Supplementary data file 2**. *SD2_phyloPred_lambda.totalfat.csv*. Predicted total fat content for Actinopterygii species.
- **Supplementary data file 3**. *SD3_phyloPred_lambda.omega3.csv*. Predicted omega-3 fatty acids content for Actinopterygii species.
- **Supplementary data file 4**. *SD4_phyloPred_lambda.omega6.csv*. Predicted omega-6 fatty acids content for Actinopterygii species.
- **Supplementary data file 5**. *SD5_phyloPred_lambda.iron.csv*. Predicted iron content for Actinopterygii species.
- **Supplementary data file 6**. *SD6_phyloPred_lambda.zinc.csv*. Predicted zinc content for Actinopterygii species.
- **Supplementary data file 7**. *SD7_phyloPred_lambda.vitA.csv*. Predicted vitamin A content for Actinopterygii species.
- **Supplementary data file 8**. *SD8_phyloPred_lambda.vitB12.csv*. Predicted vitamin B12 content for Actinopterygii species.
- **Supplementary data file 9**. *SD9_phyloPred_lambda.vitD.csv*. Predicted vitamin D content for Actinopterygii species.
- **Supplementary data file 10**. *SD1_phyloPred_lambda.protein.csv*. Predicted protein content for Actinopterygii species.
- **Supplementary data file 11.** *SD11_bionut.evolCorMat.R.* Script for evolutionary correlations.
- **Supplementary data file 12.** *SD12_bionut.PGLS.R.* Script for phylogenetic least squares regression.
- **Supplementary data file 13.** *SD13_phylosigByVar.R.* Script for estimating phylogenetic signal of nutrient variables.
- **Supplementary data file 14.** *SD14_predValidation.lambda-only.R.* Script for carrying out validation for predictions under the lambda model.
- **Supplementary data file 15.** *SD15_predValidation.pgls-lambda.R.* Script for carrying out validation for predictions under the lambda plus phylogenetic regression model.
